# Theoretical Investigation of Quantum Size Effect on the Electronic Structure and Photoelectric Properties for Graphdiyne Nanotubes

**DOI:** 10.3390/nano15161219

**Published:** 2025-08-09

**Authors:** Tao Zhang, Hanbo Wen, Zhou Li, Xinyu Zhao, Xiaoming Wang, Jingang Wang

**Affiliations:** 1 Changchun Institute of Optics, Fine Mechanics and Physics, Chinese Academy of Sciences, Changchun 130033, China; 2 College of Science, Liaoning Petrochemical University, Fushun 113001, China

**Keywords:** quantum size effect, graphdiyne nanotubes, absorption, bandstructure, charge difference density

## Abstract

In this paper, the electronic structure and photoelectric properties of graphdiyne nanotubes with armchair (A-GDYNT) and zigzag (Z-GDYNT) types have been studied. Calculations show that as n decreases, the divergence in gap values between (n)-A-GDYNT and (n)-Z-GDYNT increases. This is mainly attributed to the edge effect arising from their different boundaries. Plasmon spectra are generated in all three directions of X, Y, and Z, with the spectra along the Z direction being more prominent. The optical absorption process exhibits not only the nonlinear nature of the GDYNTs, but also a good regularity, especially in the infrared region. As the pore size increases, the A-GDYNT and Z-GDYNT exhibit striking differences in how their charge self-organizes. Likewise, notable distinctions emerge in the evolutionary pattern of their charge difference density under excitation. The porous structure and excellent sorption ability in various light regions make GDYNTs have great potential application in the field of photocatalysis and far infrared detection.

## 1. Introduction

Graphyne is an emerging carbon nanomaterial and the first new carbon allotrope formed with two hybridization states, sp and sp^2^. Graphyne is also a Dirac cone material, which is an important reason why graphyne with a band gap surpasses zero-band-gap graphene in many properties [1,2,3,4,5,6]. Although two-dimensional graphdiyne nanoribbons and graphene are both van der Waals materials [7], the former possess an intrinsic band gap and exhibit typical semiconductor characteristics. Moreover, various graphdiyne nanotubes (GDYNT) can be formed by cutting graphdiyne nanoribbons along different directions at varying sizes and subsequently curling them. Based on the quantum size effect and the boundary effect, GDYNT also exhibits physical properties that are different from those of two-dimensional graphyne [8]. Graphdiyne has a natural band gap, endowing GDYNT with typical semiconductor characteristics. Besides double bonds, there are also triple bonds in its two-dimensional framework. These special chemical bond structures make it easier for electrons to be transported within the material, thus demonstrating relatively high electrical conductivity [9]. Carriers can move rapidly in graphdiyne, enabling graphdiyne to respond to electric current more quickly [10]. Nanotubular structures are highly beneficial for regulating the electronic properties of the system [11]. Therefore, if graphdiyne is curled into quasi-one-dimensional nanotubes, due to its tubular structure and curvature effect, it will have a further impact on the electronic structure [12]. Under the action of an electric field, GDYNT will exhibit field-effect characteristics, that is, their resistance or conductance will change with the variation in the applied electric field [13]. Graphdiyne exhibits unique electronic structures and chemical bonding characteristics. In the visible to near-infrared range, GDYNT demonstrates relatively strong optical absorption capabilities, enabling it to absorb light of specific wavelengths [14]. When excited by light of an appropriate wavelength, electrons undergo a transition from the ground state to the excited state. Subsequently, as these electrons return from the excited state to the ground state, they release energy in the form of fluorescence. The emission wavelength and intensity of fluorescence are related to factors such as the structure, size of GDYNT, and the surrounding environment [15]. An important indicator for measuring the strength of the fluorescence-emitting ability of GDYNT is the quantum yield. By means of changing the preparation methods, doping elements, surface modifications, etc., of GDYNT [16]. The photoluminescence quantum yield of it can be regulated. A higher quantum yield means that the material can convert the absorbed light energy into fluorescence emission more effectively. This controllability enables researchers to design and prepare GDYNT materials with different optical properties according to specific application requirements. Owing to the unique edge structures of graphdiyne nanoribbons, they can be transformed into either armchair or zigzag configurations through different cutting approaches. This structural transformation, in turn, results in alterations to their photoelectric properties. The special structure of GDYNT endows it with potential application prospects in new energy, superconductivity, and other fields. However, up to now, no experiments have been conducted to carry out theoretical research on the plasmons of two-dimensional graphdiyne. In terms of thermodynamics, GDYNT exhibits notable properties. These include low thermal conductivity [17,18,19,20,21], negative thermal expansion, good thermal stability, and thermal responsiveness. In terms of magnetism, GDYNT possesses distinct characteristics as well. These features include weak intrinsic magnetism, doping-induced magnetism, and spin-polarization effects. In addition, GDYNT also has size-dependent effects such as energy–gap variation, change in electronic density of states, and alteration in electronic energy levels near the Fermi level [22,23].

Notably, the periodic porous structure and edge configurations of GDYNTs enable tailored infrared absorption in critical bands (3–5 μm and 8–12 μm), aligning with atmospheric transmission windows and thermal imaging detection ranges. Armchair GDYNTs with smaller pores exhibit stronger mid-wave infrared (MWIR) absorption due to quantum confinement, while zigzag GDYNTs dominate in long-wave infrared (LWIR) regimes owing to extended π-conjugation. Combined with their low thermal conductivity and negative thermal expansion [17,18], such wavelength-selective absorption positions GDYNTs as promising candidates for infrared stealth coatings, effectively suppressing thermal radiation signatures in surveillance-relevant bands.

Currently, theoretical and experimental research on GDYNT primarily focuses on computational studies using density functional theory. These calculations investigate key photoelectric properties, including electronic structure, density of states, and charge transport [24]. Through theoretical simulation, the mechanical properties such as elastic modulus and hardness are analyzed [25]. In addition, there is research on its adsorption performance through theoretical calculations. Experimentally, GDYNTs are mainly synthesized under certain conditions through chemical reactions such as the cross-coupling reaction of alkynes [22]. Most of the physical synthesis methods are still in the laboratory testing stage and have not been mass-produced on a large scale. Experimentally, X-ray diffraction (XRD) is also used to analyze its crystal structure as well as the existence of carbon–carbon single bonds and carbon–carbon triple bonds [26]. And the electrical properties of GDYNT are tested, such as measuring their resistance, conductance, and carrier mobility [27].

GDYNT has quite promising application prospects in many fields. In solar cells, GDYNTs are capable of enhancing the photoelectric conversion efficiency of solar cells. In fuel cells, it can improve the performance and stability of fuel cells [28]. By taking advantage of the sensitive characteristics of GDYNT to gas molecules, humidity, etc., sensors and other devices can be fabricated [29]. Currently, the theoretical research on the photoelectric characteristics of GDYNT with different pore sizes is still not quite extensive. Research is being conducted on the photoelectric characteristics of GDYNT with varying pore sizes under the quantum size effect. Additionally, explanations of their underlying physical mechanisms will provide valuable theoretical foundations. Such insights will also play a promotional role in advancing the application of GDYNT in the photoelectric field of micro-nano devices.

## 2. Materials and Methods

In this work, the QuantumATK−2019 package (Fermitech, Beijing, China) [30] was used to calculate the photoelectric characteristics of quasi-one-dimensional GDYNT with different sizes and two different edge structures (armchair type and zigzag type). The basis set was a linear combination of atomic orbitals (LCAO), and pseudopotentials were determined by Pseudo Dojo [31]. The electronic exchange–correlation potential was treated by the generalized gradient-Perdew Burke Ernzerh (GGA-PBE), combined with the density functional theory with Grimme’s D3 correction and without damp (DFT-D3) dispersion correction; DFT-D3 can better perform van der Waals correction [32]. As a widely used generalized gradient approximation (GGA) functional, GGA-PBE exhibits stable performance in describing the geometric structures and basic electronic properties of nanomaterials. It also features moderate computational cost, making it suitable for the size requirements of the one-dimensional nanotube systems in our study. Weak van der Waals forces exist in the stacking or interfacial interactions of nanotubes. Grimme’s D3 correction can effectively compensate for the deficiency of GGA functionals in describing long-range dispersion interactions, thereby improving the accuracy of structural optimization and energy calculation [33]. In the calculations, the DFT-LCAO module is employed with a 55-Hartree cut-off energy, which combines the pseudopotential with the linear combination of atomic orbitals. The convergence criterion for the total energy is set at 10^−5^ eV, and the *K*-mesh was 1 × 1 × 3. Among them, the armchair GDYNTs are formed by curling along the $\vec{n}$ direction, while the zigzag GDYNTs are formed by curling along the $\vec{m}$ direction (Figure S1). GDYNT with different structures and pore sizes can be obtained by curling graphdiyne nanoribbons of different lengths through cutting. Among them, the lattice constants of different A-GDYNT are a= 16.0161 Å, 19.0241 Å, 22.0321 Å, 25.0401 Å, 28.0482 Å, 31.0562 Å, respectively, and c = 16.3679 Å. The lattice constants of Z-GDYNT are a = 20.4201 Å, 25.6302 Å, 30.8402 Å, 36.0503 Å, 41.2603 Å, and 46.4704 Å, respectively, and c = 9.45 Å.

The dielectric constant, which reflects the extent of a material’s response to an electric field, can be expressed as a complex number.

(1) $$\varepsilon \left(\omega \right)=\varepsilon_{1}\left(\omega \right)+i\varepsilon_{2}\left(\omega \right)$$

In the equation, ω is the angular frequency of the external electromagnetic radiation, and $\varepsilon_{1}$ and $\varepsilon_{2}$ are the real part and the imaginary part, respectively. Basically, $\varepsilon_{1}$ represents the phase modulation or dispersion, which is the photon emission from the excited state to the steady state, and $\varepsilon_{2}$ denotes the modulation of amplitude or energy loss, corresponding to the process of electronic transitions after the resonant absorption.

The absorption coefficient is defined as follows:

(2) $$\alpha =2\frac{\omega }{c}k$$

In the formula, k is the extinction coefficient, and c is the light velocity in the vacuum.

## 3. Results

Taking a single unit cell of graphdiyne as n, GDYNT with different structures and pore sizes can be rolled out by increasing the number of unit cells. The specific pore size can be obtained by measuring the optimized structure.

**Figure 1:**
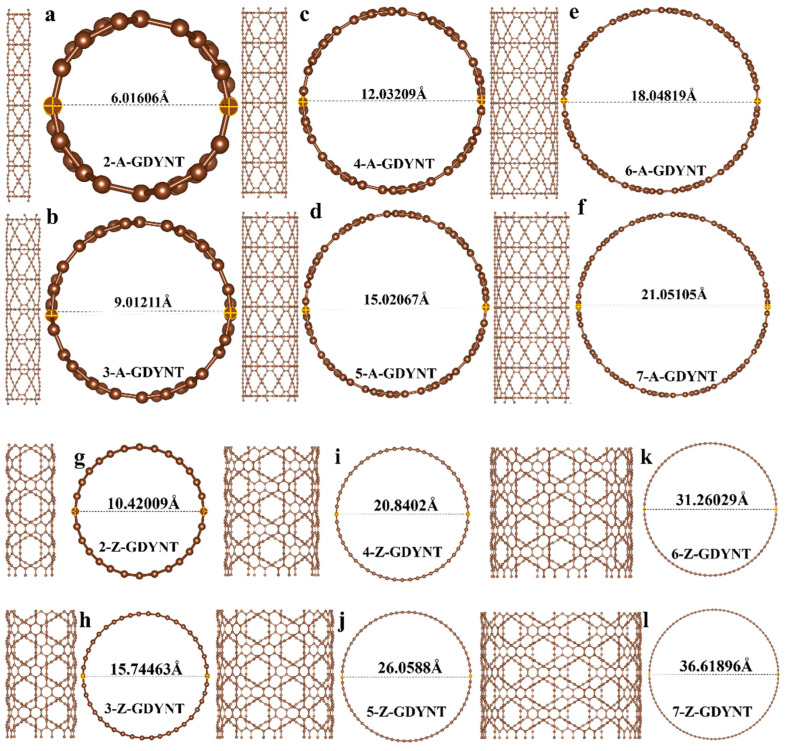
(**a**–**f**) Structure and pore sizes of (n)-A-GDYNT, *n* = 2~7. (**g**–**l**) Structure and pore sizes of (n)-Z-GDYNT, *n* = 2~7.

It can be seen that when *n* = 2, the pore diameters of the A-GDYNT and Z-GDYNT are approximately 6.016 Å (Figure 1a) and 10.420 Å (Figure 1g), respectively. When *n* = 7, their pore diameters are approximately 21.051 Å (Figure 1f) and 36.619 Å (Figure 1l), respectively. Therefore, both A-GDYNT and Z-GDYNT show increasing pore diameters as *n* increases; however, the increment in pore diameter is larger for Z-GDYNT than for A-GDYNT.

**Figure 2:**
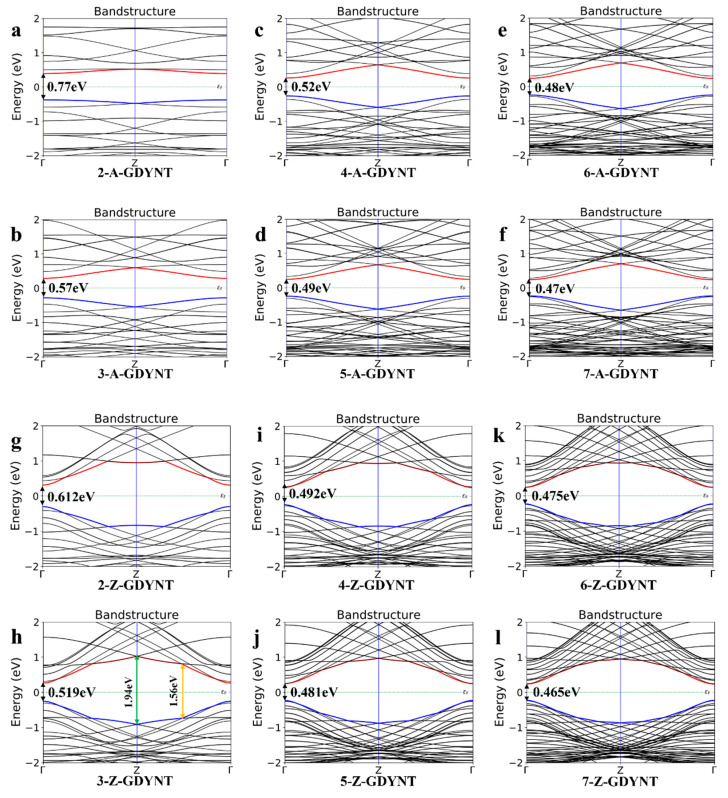
(**a**–**f**) Electron band structure of (*n*)-A-GDYNT, *n* = 2~7. (**g**–**l**) Electron band structure of (*n*)-Z-GDYNT, *n* = 2~7. The red line is CB, and the blue line is VB.

The electronic structure of a material encompasses its energy band and the density of states (DOS) at the Fermi level. This structure governs many key physical properties, including conductivity and thermoelectric characteristics. Combined theoretical and experimental research has revealed an intricate and subtle relationship between the electronic structure and the microstructure of a given material. Thus, the energy bands for (n)-GDYNT (*n* = 2~7) were computed and shown in Figure 2.

It can be seen that when *n* = 2, the band gap of the A-GDYNT is 0.77 eV (Figure 2a). As n increases continuously, the ground state energy at the bottom of the conduction band (depicted by the red line in Figure 2) decreases gradually. Meanwhile, the ground state energy at the top of the valence band (shown by the blue line in Figure 2) rises. When n is increased to 7 (Figure 2f), its band gap has reduced to 0.47 eV. Thus, the band gap of the A-GDYNT becomes narrower and narrower with the rise in *n*.

Similarly, when *n* = 2 (Figure 2g), the band gap of the Z-GDYNT is 0.612 eV; as n increases consecutively, the ground state energy at the conduction band bottom—represented by the red line in Figure 2—decreases gradually. In contrast, the ground state energy at the valence band top—indicated by the blue line in Figure 2—rises. When n is increased to 7 (Figure 2l), the band gap has decreased to 0.465 eV. Thus, the band gap of the Z-GDYNT becomes narrower and narrower with the increase in *n*.

**Figure 3:**
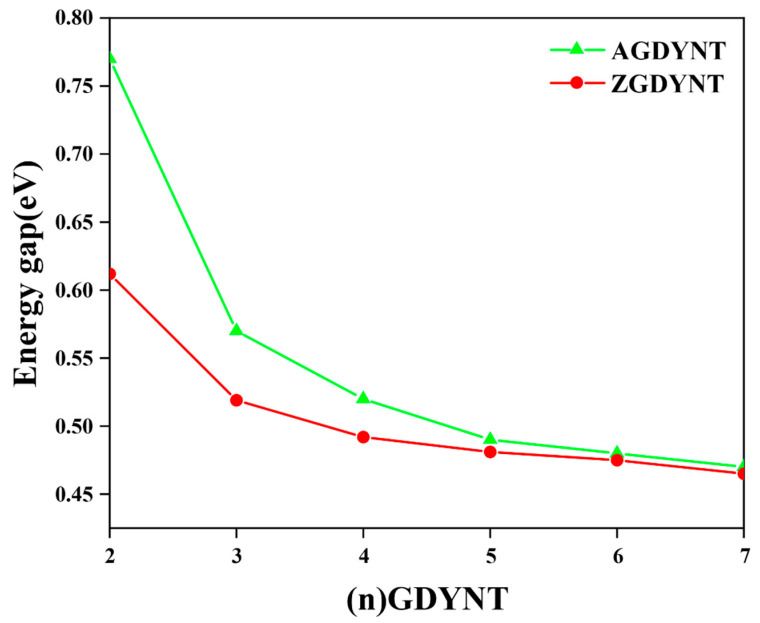
Variation in the band gap of A-GDYNT and Z-GDYNT with *n*.

Collectively, the n dependence of the band gap could be summarized and illustrated in Figure 3. As can be seen in Figure 3, the values of the band gap, whether for A-GDYNT or Z-GDYNT, drop as *n* increases monotonically. However, the initial changing rate seems to be much more sensitive to n for the A-GDYNT. When *n* = 2, the band gaps of A-GDYNT and Z-GDYNT are 0.77 eV and 0.612 eV, respectively. By contrast, when n reaches 7, both values converge to approximately 0.47 eV. This trend indicates that the band gap of Z-GDYNT becomes more stable as n increases. At *n* = 7, the band gap value is also close to that of graphdiyne nanoribbons without applied stress [2]. This similarity verifies the reliability of our calculation results. The reason lies in the fact that when n increases to a certain extent—even approaching infinity—quantum shape effects weaken and eventually disappear. As a result, the properties and results of GDYNT should exhibit a certain degree of consistency with those of graphdiyne nanoribbons.

The photo-induced electron transfer (PET) is the most fundamental photochemical process. After being excited by light, an electron transfer occurs between an excited electron donor and an acceptor, forming an intramolecular charge transfer (ICT) state. Charge difference density serves as a key method for exploring electronic structures.

**Figure 4:**
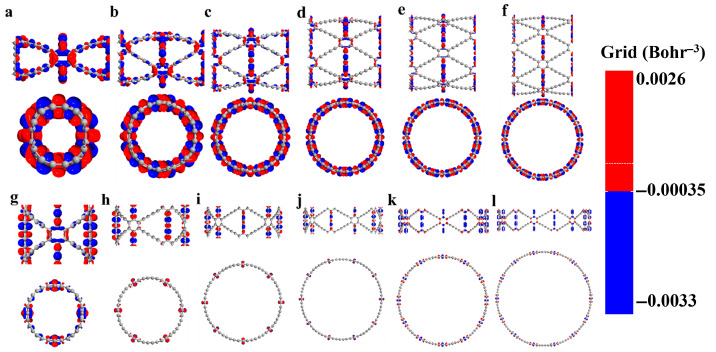
(**a**–**f**) Charge difference density of (*n*)-A-GDYNT, *n* = 2~7. (**g**–**l**) Charge difference density of (*n*)-Z-GDYNT, *n* = 2~7. Red represents electrons and blue represents holes. The isosurface value is set to 0.0003.

It reveals the direction and outcome of charge transport. This allows for an intuitive understanding of electron flow and changes in charge density within the molecule. Moreover, it facilitates the exploration of the nature of chemical bonds. To investigate photochemical effects, infrared radiation with energy matching their band gaps was used to excite the GDYNTs. The charge difference density differences of (*n*)-GDYNT, as shown in Figure 4, allow for visual analysis of electron and hole transfer.

In these maps, red is electrons and blue denotes holes. From *n* = 2 (Figure 4a) to *n* = 7 (Figure 4f), a consistent pattern emerges in A-GDYNT: most electron-hole transfer occurs along the longitudinal −C≡C−C≡C− linkages connecting benzene rings. A smaller portion of this transfer takes place at the transverse −C≡C−C≡C− groups, which connect benzene rings to one another. Under such circumstances, electrons mainly accumulate on the carbon–carbon single bonds, while holes are present on the carbon–carbon triple bonds. When n exceeds 5, charge transfer weakens noticeably—both on the benzene rings and along the longitudinal −C≡C−C≡C− chains. By *n* = 6 and 7, charge transfer in the carbon chains disappears entirely. Only a small amount of electron and hole distribution remains on the transverse −C≡C−C≡C− chains.

The curvature along the Z direction on the surface of the GDYNT is zero. Under the lower energy excitation, a relatively large amount of charge transfer is not expected in the longitudinal carbon chain. However, when graphdiyne curls into circular rings, quantum size and shape effects come into play. These effects lead to large-scale charge redistribution on transverse carbon chains—and especially on benzene rings. This phenomenon is particularly pronounced for small values of n, such as *n* = 2 and 3. In turn, this redistribution causes synchronous charge transfer in the longitudinal carbon chains. As *n* increases and the diameter of GDYNT enlarges, not only the quantum effects, but also the charge redistribution on the transverse carbon chain and benzene rings diminish. Therefore, charge transfer on the longitudinal carbon chain is no longer needed either. This is the reason why as *n* rises, the massive longitudinal charge transfer lessens or even disappears eventually.

Observing the charge difference density of (*n*)-Z-GDYNT from Figure 4g to Figure 4l, a distinct pattern emerges. At *n* = 2, electron-hole transfer in Z-GDYNT primarily takes place on the transverse −C≡C−C≡C− linkages connecting benzene rings, as well as on the benzene rings themselves. Only a small number of holes remain on the longitudinal carbon chains. When *n* = 3, 4, and 5, the holes on the longitudinal chains disappear, and charge transfer only happens on transverse carbon chains. Among these structures, electrons are concentrated on the carbon–carbon single bonds, while holes are localized on the carbon–carbon triple bonds. This is partly due to the interaction between charges on carbon chains and those on benzene rings. More importantly, the triple bond contains two π bonds. These π bonds form when holes fill the molecular free electron layer. As a result, the carbon–carbon triple bond acquires a genuine positive charge affinity. When n increases continuously to 6 and 7, electron-hole transfer occurs on benzene rings and the transverse carbon chains linking them. In this scenario, electrons are consistently distributed on the benzene rings. Holes, however, are not limited to the benzene rings—they also reside on the carbon–carbon triple bonds of the transverse carbon chains. Furthermore, we have employed population analysis to quantitatively assess the amount of charge transfer. With the increase in *n*, the population of A-GDYNT in the x and y directions is significantly stronger than that in the z direction (Tables S1–S4). This further confirms our previous conclusion that the charge transfer mainly originates from the −C≡C−C≡C− moieties. For Z-GDYNT, its population in the x and y directions is more pronounced when *n* = 2; however, as *n* increases to 7, the population in the z direction becomes dominant. This indicates that the charge transfer occurs from the −C≡C−C≡C− segments linked to the benzene rings. Compared with the (*n*)-A-GDYNT, it could be observed that there is a completely different charge transfer mechanism or trend in the (*n*)-Z-GDYNT as n increases under infrared irradiation. This phenomenon may be attributed to the quantum boundary effects associated with different edge structures.

**Figure 5:**
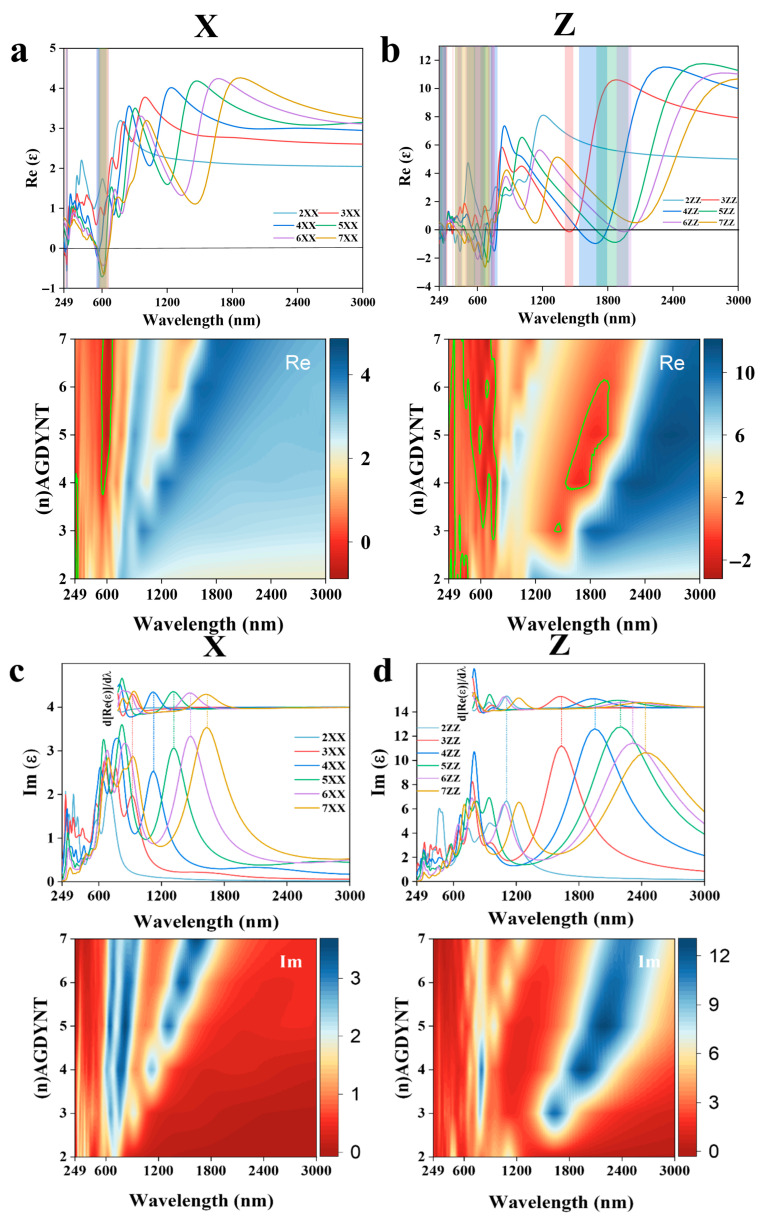

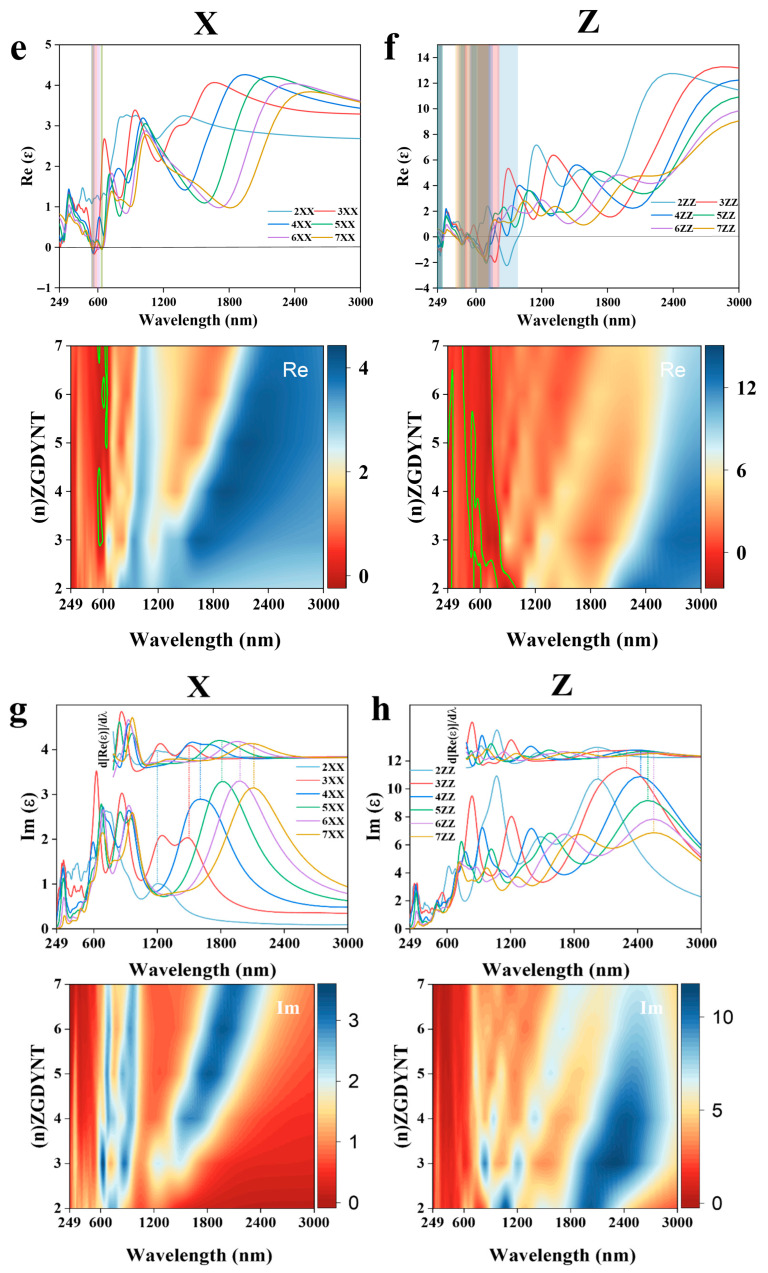
(**a**) Real part with color-filled graph of the dielectric constant for (*n*)-A-GDYNT in the X direction and Z direction (**b**). (**c**) Imaginary part with color-filled graph of the dielectric constant for (*n*)-A-GDYNT in the X direction and Z direction (**d**); the derivative of the corresponding real part is shown in the upper. (**e**) Real part with color-filled graph of the dielectric constant for (*n*)-Z-GDYNT in the X direction and Z direction (**f**). (**g**) Imaginary part with color-filled graph of the dielectric constant for (*n*)-Z-GDYNT in the X direction and Z direction (**h**); the derivative of the corresponding real part is shown in the upper.(In the green box of the figure, there are contour lines with a value of 0).

In initial calculations, we adopted a Cartesian coordinate system to maintain consistency with previous research systems, enabling direct comparison of optical property differences across nanomaterials of varying dimensions (Figure S2). Our study revealed that due to GDYNT’s structural symmetry, its optical properties in the X and Y directions are identical. Thus, only one of the X/Y directions is presented in the main text, with comparisons made against the z direction [34]. As shown in Figure 5a, in the near-infrared region, all real parts of the dielectric constant first rise slowly and then drop sharply as the wavelength decreases. In contrast, the imaginary parts (Figure 5b) rise rapidly to a peak before decreasing drastically, forming a relatively symmetrical hill-like shape. Notably, for the same n value, the peak of the imaginary part curve and the steepest drop of the real part occur at nearly the same wavelength. This anomaly reflects the stimulated electronic transitions in (*n*)-A-GDYNT upon energy absorption in the near-infrared region. Furthermore, as n increases, the peak position and the transition process shift towards the red end, indicating that A-GDYNT with a larger pore size has stronger absorption capabilities for the infrared light with longer wavelengths.

As the wavelength decreases to the visible light range around 600 nm, the imaginary parts in the X direction once again reach their local maximum, while the real parts of the dielectric constant decline to the local minimum simultaneously, especially for *n* = 4~7.

Their values even dip into the negative, a minus real part of the dielectric constant indicates that after absorbing visible light, the (*n*)-A-GDYNT (*n* = 4~7) generates plasmonic spectra, which is shown in the green frame of Figure 5a. Another plasmon production happens in the ultraviolet region in the vicinity of around 270 nm (green frame), characterizing the resonance absorption of UV by the A-GDYNTs. It seems that for the A-GDYNT, the smaller n and the pore size, the higher the frequency of electromagnetic wave is needed to generate plasmons, and the higher the plasmonic energy could be acquired.

Similarities occur in the Z direction for the dielectric constant of (*n*)-A-GDYNT, as shown in Figure 5b. Plasmonic spectra emerge in the visible and ultraviolet ranges. However, the spectral bandwidth of the generated plasmons is significantly broader than that along the X direction. For instance, in these regions (green frame), the bandwidths measure 360–750 nm and 249–300 nm, respectively. Interestingly, not only the resonance absorption as in the X axis was observed in the near-infrared region, but also the plasmons could be generated for *n* = 3~6 within the wavelength of 1350–1950 nm (green frame). Although weaker in intensity than that under visible light, the plasmonic spectra still show strong regularity and extreme stability, which is most obvious when *n* = 5, corresponding to the summit of the imaginary part for the dielectric constant at 2208 nm in Figure 5d. Compared with the X direction, the peak of the imaginary part at the same n in Figure 5d all shifts to the red end, suggesting that A-GDYNT has stronger long-wave infrared absorption ability along the Z direction.

The dielectric constant with contour map for (*n*)-Z-GDYNT is exhibited in Figure 5e–h, and it can be seen that things happen analogously with the (*n*)-A-GDYNT. In the near-infrared region, distinct resonance absorption occurs along the X direction within the wavelength range of 1200–2484 nm (Figure 5e,g). In the visible light region (558–641 nm), plasmonic spectra (green frame) are generated for *n* = 3 to 7. Notably, for *n* = 3, the peak value of the imaginary part reaches its maximum at 630 nm (Figure 5g). At the same time, the intensity of the plasmon seems much weaker than that of the (*n*)-A-GDYNT. Resonance absorption still exists in the near-ultraviolet, but surprisingly, no plasmon is produced for (*n*)-Z-GDYNT (*n* = 2~4) in the X direction around 250 nm, demonstrating the most apparent difference with the A-GDYNT.

Along the Z direction, the (*n*)-Z-GDYNT exhibits the electromagnetic interaction with the near-infrared and even the thermal infrared to a certain degree, as shown in Figure 5f,h. Noticeably, for the same n value, all peaks shift toward the red end compared to those of A-GDYNT in Figure 5d. This red shift also occurs along the X direction in Figure 5g. This phenomenon may stem from the pore size effect: for the same n, Z-GDYNT consistently has a larger diameter than A-GDYNT. The larger the diameter of GDYNT, the more significant its response to longer-wavelength electromagnetic waves. When *n* = 3, the peak value reaches its maximum at 2311 nm, indicating that the (3)-Z-GDYNT has the strongest absorptive capability for the near-infrared along the Z direction. As the wavelength decreases to the ranges of 249–303 nm and 417–984 nm, plasmonic spectra emerge for (*n*)-Z-GDYNT with n values from 2 to 7 (green frame). What’s more, the plasmons generated by (2)-Z-GDYNT can be excited not only by ultraviolet light but also by near-infrared radiation, as shown in Figure 5f. This characteristic makes (2)-Z-GDYNT differ most significantly from (2)-A-GDYNT. Generally, in the visible light and ultraviolet range, the A-GDYNT can produce plasmons much more efficiently than the Z-GDYNT, maybe due to the pore size effect. While the interaction between electromagnetic waves in different frequency bands, as well as the ability to generate plasmons, has more advantages in the Z direction than in the X direction, whether for the A-GDYNT or Z-GDYNT, this could be attributed to the shape influences. Both (*n*)-A-GDYNT and (*n*)-Z-GDYNT maintain a high degree of consistency in the XY direction.

**Figure 6:**
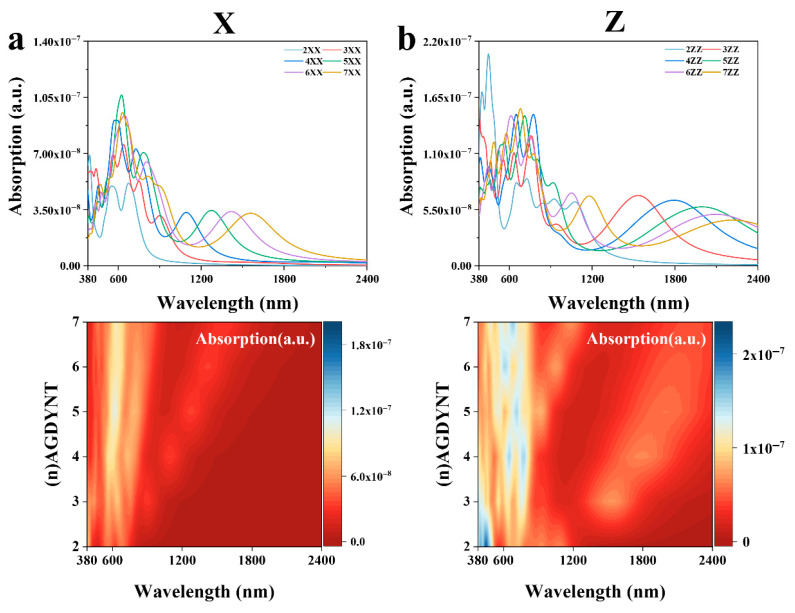

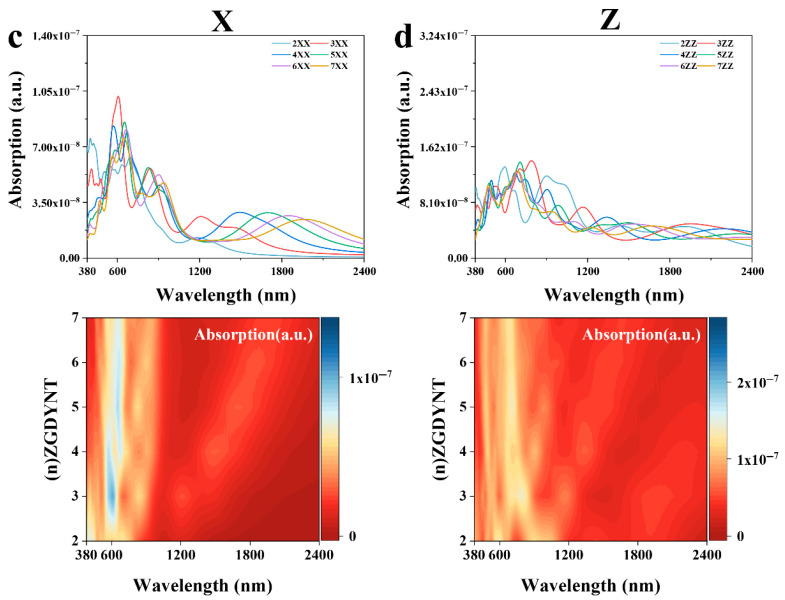
(**a**) Absorption spectra of (*n*)-A-GDYNT in the X direction. (**b**) Absorption spectra of (*n*)-A-GDYNT in the Z direction. (**c**) Absorption spectra of (*n*)-Z-GDYNT in the X direction. (**d**) Absorption spectra of (*n*)-Z-GDYNT in the Z direction.

All calculations were also performed in the X, Y, and Z directions (Figure S3). The results indicate that the properties in the X and Y directions are consistent. The absorption spectra with contour map of GDYNT in the range of 380–2400 nm are shown in Figure 6. As can be seen from Figure 6a in the X direction, the maximum excited state is at 621.18 nm for (5)-A-GDYNT, and the light absorption coefficient is $1.06\times 10^{-7}\;L/cm/mol$. Due to the good symmetry of (*n*)-A-GDYNT on the XY plane, the absorption spectra in these two directions are almost identical. Therefore, the above figure only shows the absorption spectrum of (*n*)-A-GDYNT in the X direction. In the wavelength range of 600–2400 nm, a good regularity is demonstrated, that is, with the increase in n, the peak undergoes an obvious red shift and returns to zero after 2400 nm. In the Z direction (Figure 6b), the (2)-A-GDYNT has the maximum excited state at 451.77 nm, and its light absorption coefficient reaches $2.06\times 10^{-7}\;L/cm/mol.$ From spectra curves, it can be seen that the A-GDYNT exhibits excellent nonlinear optical absorption properties under visible light.

Based on the absorption spectra of (*n*)-Z-GDYNT in the range of 380–2400 nm. In the X direction (Figure 6c), its maximum excited state is at 609.75 nm under *n* = 3, and the light absorption coefficient is $1.01\times 10^{-7}\;L/cm/mol$ In Figure 5g, the peak of the imaginary part is at 630 nm for *n* = 3, and the photon energy is 1.97 eV; such energy corresponds to the band transition symmetry points indicated by the green arrow in Figure 2h. Note that there is a tiny deviation of about 1.5% between the photon energy and the bandgap, indicating that electrons undergo relaxation during the transition process. In the wavelength range of 900–2400 nm, a good regularity is demonstrated. In the Z direction (Figure 6d), the maximum excited state is at 795.12 nm for *n* = 3, and the light absorption coefficient is $1.41\times 10^{-7}\;L/cm/mol.$ The energy for this excitation is 1.56 eV, which shows full compliance with the inter-band symmetric points indicated by the orange arrow in Figure 2h, implying that as the excitation frequency decreases, the relaxation during transition weakens. GDYNTs demonstrate excellent absorption capabilities for visible and infrared light. Their pores, which come in varying sizes, can accommodate a range of transition metal ions. Examples of such ions include Pt, Ni, and Pd. These properties make GDYNTs a promising candidate as a high-performance photocatalyst for green energy gases.

**Figure 7:**
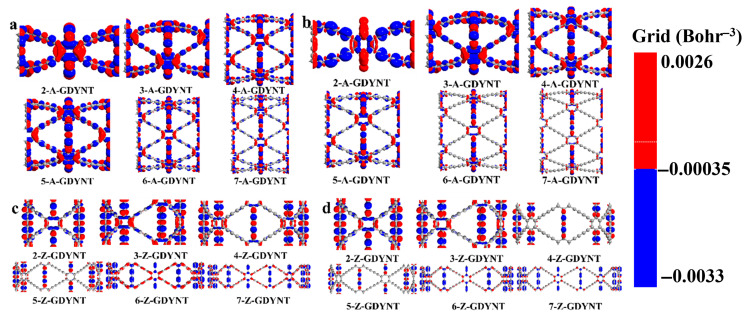
(**a**) Charge difference density irradiated by the infrared under selected wavelengths in the range of 670–1600 nm along the X direction for (*n*)-A-GDYNT. (**b**) Charge difference density irradiated by the infrared under selected wavelengths in the range of 1000–2300 nm along the Z direction for (*n*)-A-GDYNT. (**c**) Charge difference density irradiated by the infrared under selected wavelengths in the range of 1100–2000 nm along the X direction for (*n*)-Z-GDYNT. (**d**) Charge difference density irradiated by the infrared under selected wavelengths in the range of 1900–2500 nm along the Z direction for (*n*)-Z-GDYNT. Red represents electrons and blue represents holes. The isosurface value is set to 0.0003.

To examine how GDYNT responds to light in the visible and near-infrared regions, a series of electromagnetic waves was used. These waves had specific wavelengths: 671.55 nm, 903.54 nm, 1092.19 nm, 1274.23 nm, 1419.85 nm, and 1577.61 nm. They were applied to irradiate (*n*)-A-GDYNT samples with n values ranging from 2 to 7. The results of this experiment are presented in Figure 7a. These selected wavelengths correspond exactly to the absorption peak maxima of the respective (*n*)-A-GDYNT samples within the 670–1600 nm range, as shown in Figure 6a.

It can be seen in Figure 7a that for the *n*-A-GDYNT (*n* = 2~6) system, there is an alternating distribution of electrons and holes on the longitudinal, transverse carbon chains and the benzene rings. When *n* = 2~5, the isosurfaces of electrons and holes are well-distributed, indicating the local excitation on GDYNT. When *n* = 6 and 7, compared with the transverse, the amounts of electrons and holes on the longitudinal carbon chain reduce significantly, especially for electrons, implying the local excitation characteristics of the system weaken.

Figure 7b exhibits the charge difference density along the Z direction of the n-A-GDYNT (*n* = 2~7), and the incidence wavelengths are 1092.19 nm, 1529.07 nm, 1807.09 nm, 1987.8 nm, 2114.68 nm, and 2208.66 nm, respectively. It can be seen that for the 2-A-GDYNT, electrons solely concentrate on the transverse carbon chains, and on the benzene rings and longitudinal chains, electrons and holes alternate in distribution. However, the electron density on the single bonds of the longitudinal carbon chain is significantly lower than the hole density on the triple bonds. This observation indicates that charge transfer occurs between the longitudinal and transverse carbon chains. Notably, this transfer takes place through the benzene rings; for *n* = 3 and 4, electrons and holes are distributed on both the longitudinal and the transverse carbon chains with benzene rings alternately and homogenously. When n reaches 5, the charge distribution on the longitudinal carbon chain significantly decreases, and the longitudinal is dominated by holes. When n increases to 6 and 7, the charge difference phenomenon only exists on the transverse carbon chain, and is nearly invisible in the longitudinal direction, even on the benzene rings, it becomes blurred. Compared with the X direction, there is a much weaker local excitation for the A-GDYNT along the Z axis under the lower frequency infrared irradiation.

The excitation effects of (*n*)-Z-GDYNT have also been investigated, and a series of near infrared wavelengths of 1183.21 nm, 1212.07 nm, 1483.43 nm, 1713.62 nm, 1840.55 nm, and 1987.8 nm were employed to irradiate the (*n*)-Z-GDYNT (*n* = 2~7), respectively. These wavelengths correspond to the summits of the peak of the related absorption curves in Figure 6c, and we found the evolution pattern of charge difference density of Z-GDYNT, which is shown in Figure 7c, exhibits a wholly different pattern from that of the A-GDYNT. It can be seen that when *n* = 2 and 3, the electrons and holes distribute alternatively and evenly on the transverse carbon chain and benzene rings, while the longitudinal chains are mainly dominated by holes. As *n* increases to 4 and 5, the charge difference on the transverse carbon chain shows no visible change, but the charge on the benzene rings and the holes on the longitudinal chains conspicuously diminish until they disappear. When *n* continues to increase to 6 and 7, something remarkable happens: the entire Z-GDYNT appears to be recharged. Interestingly, this time the transverse carbon chains are fully occupied by holes on their carbon–carbon triple bonds. In contrast, the longitudinal chains are completely filled with electrons in their carbon–carbon single bonds. Meanwhile, the charges on the benzene rings show an alternating distribution of electrons and holes. As a consequence, with the increase in n and pore size, the charge transfer and redistribution between the transverse and longitudinal carbon chains are realized for Z-GDYNT through its benzene rings.

With regard to the Z direction, the charge difference density of Z-GDYNT demonstrates a very similar developing path to the X axis, and the excitation wavelengths of the near infrared are 1911.35 nm, 1948.82 nm, 2208.67 nm, 2311.39 nm, 2424.14 nm, and 2484.75 nm, respectively; the calculated results are shown in Figure 7d. When *n* = 2 and 3, the electrons and holes distribute alternatively and homogeneously on the transverse carbon chain and benzene rings, and the longitudinal chains are inhabited by the scattered holes. As n rises to 4 and 5, the charge difference on the transverse carbon chain shows no distinguishable change, but the charge on the benzene rings and the holes on the longitudinal chains significantly decrease until they vanish. When *n* increases further to 6 and 7, the entire Z-GDYNT becomes recharged again. At this point, holes are located on the transverse carbon chains, while electrons accumulate on the longitudinal chains. However, the number of electrons here appears smaller than in the X-direction scenario shown in Figure 7c. Similarly, the charges on the benzene rings exhibit an alternating distribution of electrons and holes.

Comparing the consequences of charge difference density between A-GDYNT and Z-GDYNT under near-infrared excitation, just as our description above, it can be witnessed that they have remarkable differences in their charge self-organization or evolutionary manner. This is mainly determined by their structure type, for example, the armchair or the zigzag nature. Meanwhile, the energy of the infrared photons used for excitation may also have a partial impact on the charge difference results to some extent.

## 4. Conclusions

In summary, the first-principles calculation was employed to conduct a theoretical investigation on the electronic structures and optical properties of GDYNT with different pore diameters and structures under the quantum size effect and the boundary effect.

After structural optimization, their band structures were calculated. It can be concluded that as n increases, the band gap for A-GDYNT and Z-GDYNT both decrease monotonically, while the variation in the gap value is much more sensitive for the A-GDYNT at small n, suggesting the edge structures give influences on the electronic structures of GDYNT. When n increases to 7, due to the weakening of the quantum shape effect, the gap values of these two types of GDYNT tend to be identical. From the charge difference density maps, it can be seen that the electron-hole transfer in the A-GDYNT mainly occurs on the −C≡C−C≡C− that links the benzene rings longitudinally; by contrast, the electron-hole transfer of the Z-GDYNT mainly occurs on the −C≡C−C≡C− that links the benzene rings transversely.

From the dielectric constants of the A-GDYNT and Z-GDYNT, it can be seen that they both demonstrate good absorption ability in the near-infrared, visible light, and ultraviolet regions, where the electronic transition happens or plasmons are generated, and the spectral bandwidth of plasmons produced in the Z direction is much larger than that along the X direction. By analyzing the infrared spectra, it can be found that both the A-GDYNT and the Z-GDYNT show obvious regularities in the near-infrared range, that is, the peaks gradually shift to the red end with the increase in n. Also, compared with the A-GDYNT, whether along X or Z direction, all peaks under the same n move to the red end for the Z-GDYNT in the wavelength range of 2000–2600 nm, which may be due to the quantum pore size effect.

The absorption spectra exhibit not only the excellent nonlinear optical absorption effect for the GDYNTs under visible light, but also a good regularity in the wavelength range of 600–2400 nm. And as the excitation energy, i.e., the photon frequency, decreases, the relaxation during the absorption transition weakens.

When GDYNTs are excited by the energy at the absorption peak in the infrared region, the local excitation of A-GDYNTs weakens as n increases. On the other hand, there exists a remarkable discrepancy in the charge redistribution and evolutionary pattern of the charge difference density between A-GDYNT and Z-GDYNT, which is mainly due to their different structure types with various boundary effects. As the number of n increases, the charge transfer and redistribution between the transverse and longitudinal carbon chains are realized in Z-GDYNT through its benzene rings.

## 5. Patents

This section is not mandatory but may be added if there are patents resulting from the work reported in this manuscript.

## Data Availability

The original contributions presented in this study are included in the article/Supplementary Material. Further inquiries can be directed to the corresponding authors.

## Supplementary Materials

The following supporting information can be downloaded at: https://www.mdpi.com/article/10.3390/nano15161219/s1, Figure S1. The graphdiyne nanosheets with different edge structures can be obtained by cutting graphdiyne nanoribbons along different chiral directions (n, m). Curling them can further yield graphdiyne nanotubes with different edge structures. Figure S2. (a) The real and imaginary parts of the dielectric constant of A-GDYNT in the X, Y, and Z directions. (b) The real and imaginary parts of the dielectric constant of Z-GDYNT in the X, Y, and Z directions. Figure S3. Comparison diagram of absorption spectra of A-GDYNT and Z-GDYNT in the X, Y, and Z directions. Table S1. Population analysis of 7Z-GDYNT(The complete data can be found in the supplementary document (Data S1), which includes the numbering and corresponding population of all 252 C at-oms.). Table S2. Population analysis of 7A-GDYNT(The complete data can be found in the supplementary document (Data S2), which includes the numbering and corresponding population of all 252 C at-oms). Table S3. Population analysis of 2Z-GDYNT(The complete data can be found in the supplementary document (Data S3), which includes the numbering and corresponding population of all 72 C at-oms). Table S4. Population analysis of 2A-GDYNT(The complete data can be found in the supplementary document (Data S4), which includes the numbering and corresponding population of all 72 C at-oms).
