# Supplementary material for: Theoretical Investigation of Quantum Size Effect on the Electronic Structure and Photoelectric Properties for Graphdiyne Nanotubes
Source: Nanomaterials (Basel). 2025 Aug 9;15(16):1219. doi: 10.3390/nano15161219 (PMC12388027; doi:10.3390/nano15161219)
Supplement: Supplementary file 1 [file nanomaterials-15-01219-s001.zip › nanomaterials-3770823-SI-done-1.pdf]

nanomaterials

Sup Information

Theoretical Investigation of Quantum Size Effect on the Electronic Structure and Photoelectric Properties for Graphdiyne Nanotubes

Tao Zhang [1], Hanbo Wen [2], Zhou Li [1,*], Xinyu Zhao [1], Xiaoming Wang [1] and Jingang Wang [2,*]

[1] Changchun Institute of Optics, Fine Mechanics and Physics, Chinese Academy of Sciences, Changchun 130033, China
[2] College of Science, Liaoning Petrochemical University, Fushun 113001, China
* Correspondence: 15500027661@163.com (Z. Li); jingang_wang@lnpu.edu.cn (J. Wang)

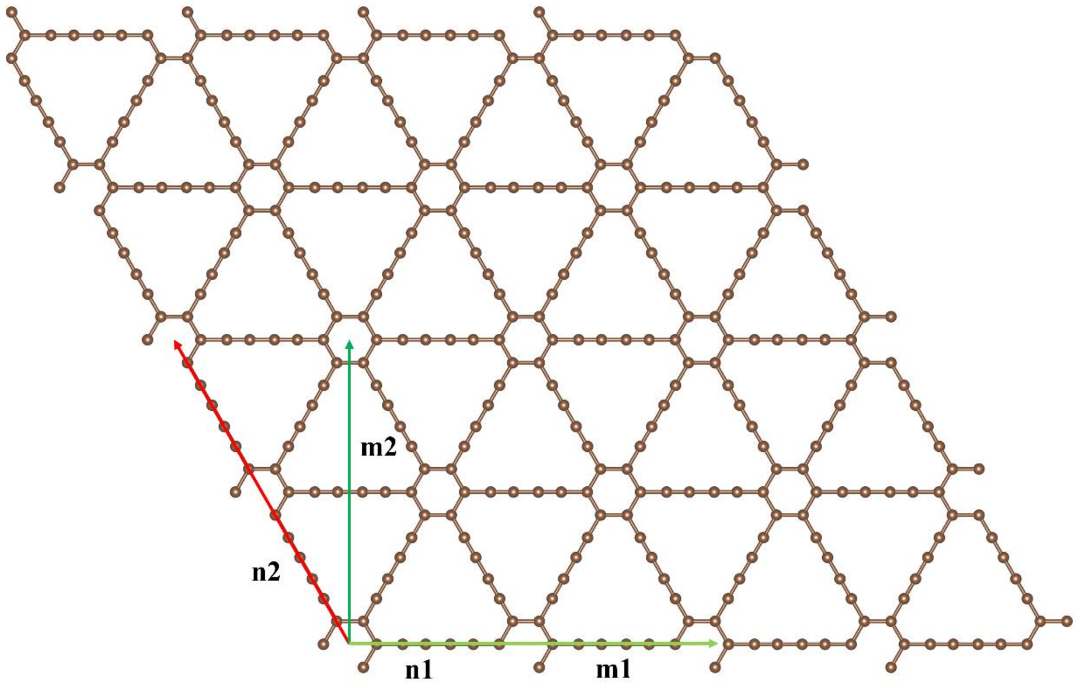

Figure S1. The graphdiyne nanosheets with different edge structures can be obtained by cutting graphdiyne nanoribbons along different chiral directions (n, m). Curling them can further yield graphdiyne nanotubes with different edge structures.

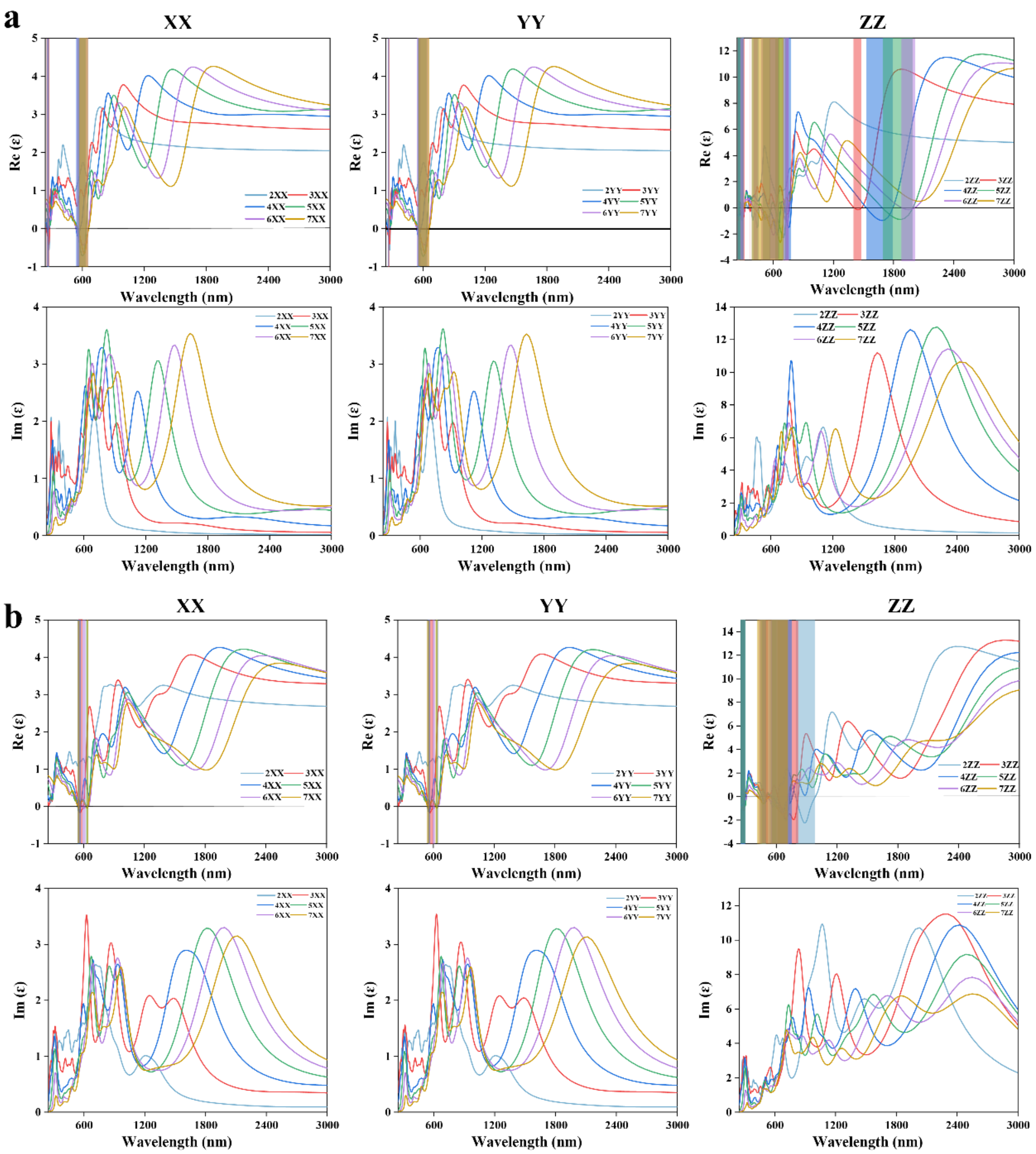

Figure S2. (a) The real and imaginary parts of the dielectric constant of A-GDYNT in the X, Y, and Z directions. (b) The real and imaginary parts of the dielectric constant of Z-GDYNT in the X, Y, and Z directions.

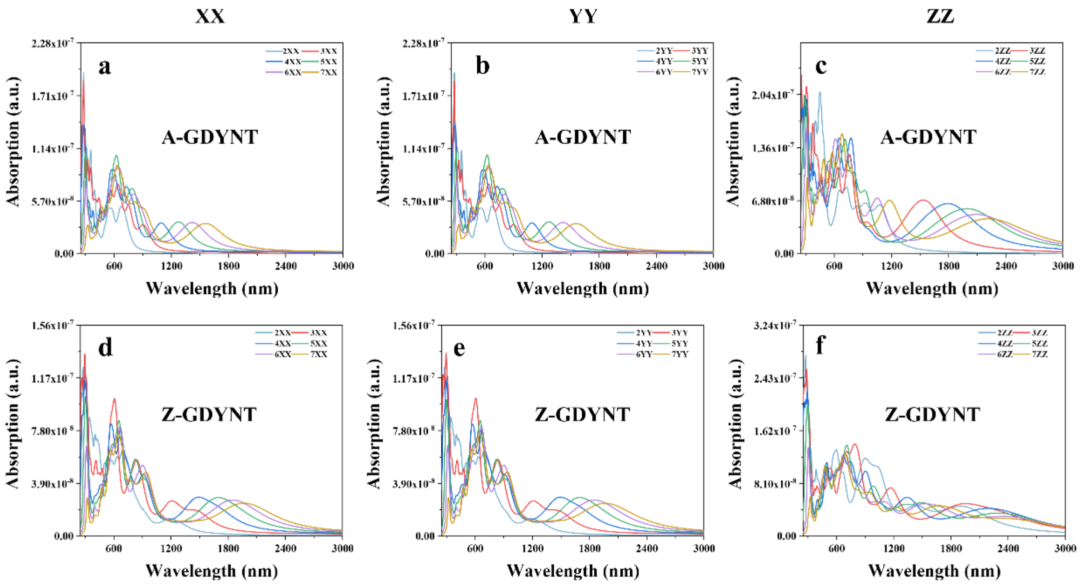

Figure S3. Comparison diagram of absorption spectra of A-GDYNT and Z-GDYNT in the X, Y, and Z directions.

Table S1. Population analysis of 7Z-GDYNT(The complete data can be found in the supplementary document (Data S1), which includes the numbering and corresponding population of all 252 C at-oms.).

Population classification	Atomic number	Total population	s-orbital population	Total population of p orbitals and hybrid orbitals
3.977~3.979	0、1、11、13、35	3.978±0.001	0.699	3.279±0.001
4.001~4.002	4、5、16、17、40	4.001±0.001	0.707	3.294±0.001
4.020~4.021	2、3、8、9、26	4.020±0.001	0.726	3.294±0.001

Table S2. Population analysis of 7A-GDYNT(The complete data can be found in the supplementary document (Data S2), which includes the numbering and corresponding population of all 252 C at-oms).

Population classification	Atomic number	Total popu-lation	s-orbital population	Total population of p orbitals and hy-brid orbitals
3.977~3.979	0、5、11、15、20、25、35、40	3.978±0.001	0.700	3.278±0.001
4.000~4.001	2、3、8、9、16、17、22、27	4.000±0.001	0.707	3.293±0.001
4.020~4.024	1、4、7、10、12、19、23、28	4.022±0.002	0.726	3.296±0.002

Table S3. Population analysis of 2Z-GDYNT(The complete data can be found in the supplementary document (Data S3), which includes the numbering and corresponding population of all 72 C at-oms).

Population classification	Atomic number	Total popula-tion	s-orbital popula-tion	Total population of p orbitals and hy-brid orbitals

3.977~3.978	0、1、10、11、22、23、34、35	3.977±0.001	0.700	3.277±0.001
3.998~4.000	4、5、6、7、16、17、40、41	3.999±0.001	0.707	3.292±0.001
4.021~4.026	2、3、8、9、14、15、32、33	4.024±0.002	0.725	3.300±0.003

Table S4. Population analysis of 2A-GDYNT(The complete data can be found in the supplementary document (Data S4), which includes the numbering and corresponding population of all 72 C at-oms).

Population classification	Atomic number	Total population	s-orbital population	Total population of p orbitals and hybrid orbitals
3.977~3.979	0、5、11、15、20、25、35、40	3.978±0.001	0.700	3.278±0.001
4.000~4.001	2、3、8、9、16、17、22、27	4.000±0.001	0.707	3.293±0.001
4.020~4.024	1、4、7、10、12、19、23、28	4.022±0.002	0.726	3.296±0.002
